# Association of Aortic Root and Valve Morphology With De Novo Aortic Valve Regurgitation After Implantation of Left Ventricular Assist Device

**DOI:** 10.1111/aor.14987

**Published:** 2025-03-12

**Authors:** Takashi Murakami, Yusuke Misumi, Daisuke Yoshioka, Takuji Kawamura, Ai Kawamura, Shin Yajima, Shunsuke Saito, Takashi Yamauchi, Shigeru Miyagawa

**Affiliations:** ^1^ Department of Cardiovascular Surgery Osaka University Graduate School of Medicine Suita Osaka Japan

**Keywords:** continuous‐flow left ventricular assist device, de novo aortic valve insufficiency, aortic root morphology

## Abstract

**Background:**

The development of aortic valve regurgitation (AR) negatively affects the survival of patients with continuous‐flow left ventricular assist device (LVAD) support. Although several risk factors have been identified, little is known about the effect of preoperative aortic root and valve morphology on the development of de novo AR after LVAD implantation.

**Methods:**

Between April 2018 and September 2023, 87 patients underwent durable LVAD implantation at our department. Of these, the 15 eligible patients who underwent preoperative electrocardiography‐synchronized cardiac contrast‐enhanced computed tomography were included in this study. Baseline aortic root and valve morphology and its relationship with the postoperative development of AR were retrospectively reviewed.

**Results:**

The mean duration of LVAD support was 1208 ± 618 days. At 60 months postsurgery, 10 patients had mild or greater AR (Group I) and the others did not (Group N). The measurement of baseline aortic root morphology showed that the ratio of virtual basal ring diameter to geometric height (VBD/GH) was significantly larger for Group I (1.70 ± 0.024 vs. 1.48 ± 0.034; *p* = 0.0001).

**Conclusions:**

A large preoperative VBD/GH is a significant risk factor for de novo AR. This finding may assist in determining the surgical indications for concomitant aortic valve procedures with durable LVAD implantation.

## Graphical abstract

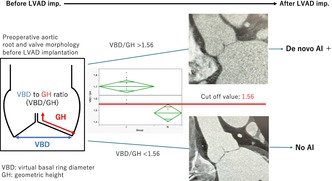

Large preoperative aortic virtual basal ring diameter to geometric height ratio may be a novel risk factor for de novo aortic insufficiency after durable LVAD implantation and assist in determining surgical indications for concomitant aortic valve procedures with LVAD implantation.

## 1 Introduction

De novo aortic regurgitation (AR) after left ventricular assist device (LVAD) implantation is one of the most serious complications associated with morbidity and mortality [1, 2]. De novo AR develops after LVAD implantation in many patients, even without preoperative AR or only with trivial AR [1]. Several investigators have reported the risk factors for de novo AR under LVAD support, but few studies have examined the relationship between aortic root morphology and de novo AR [3]. This study aimed to determine the relationship between the occurrence of de novo AR and the detailed preoperative morphology of the aortic root and valve.

## 2 Materials and Methods

**FIGURE 1:**
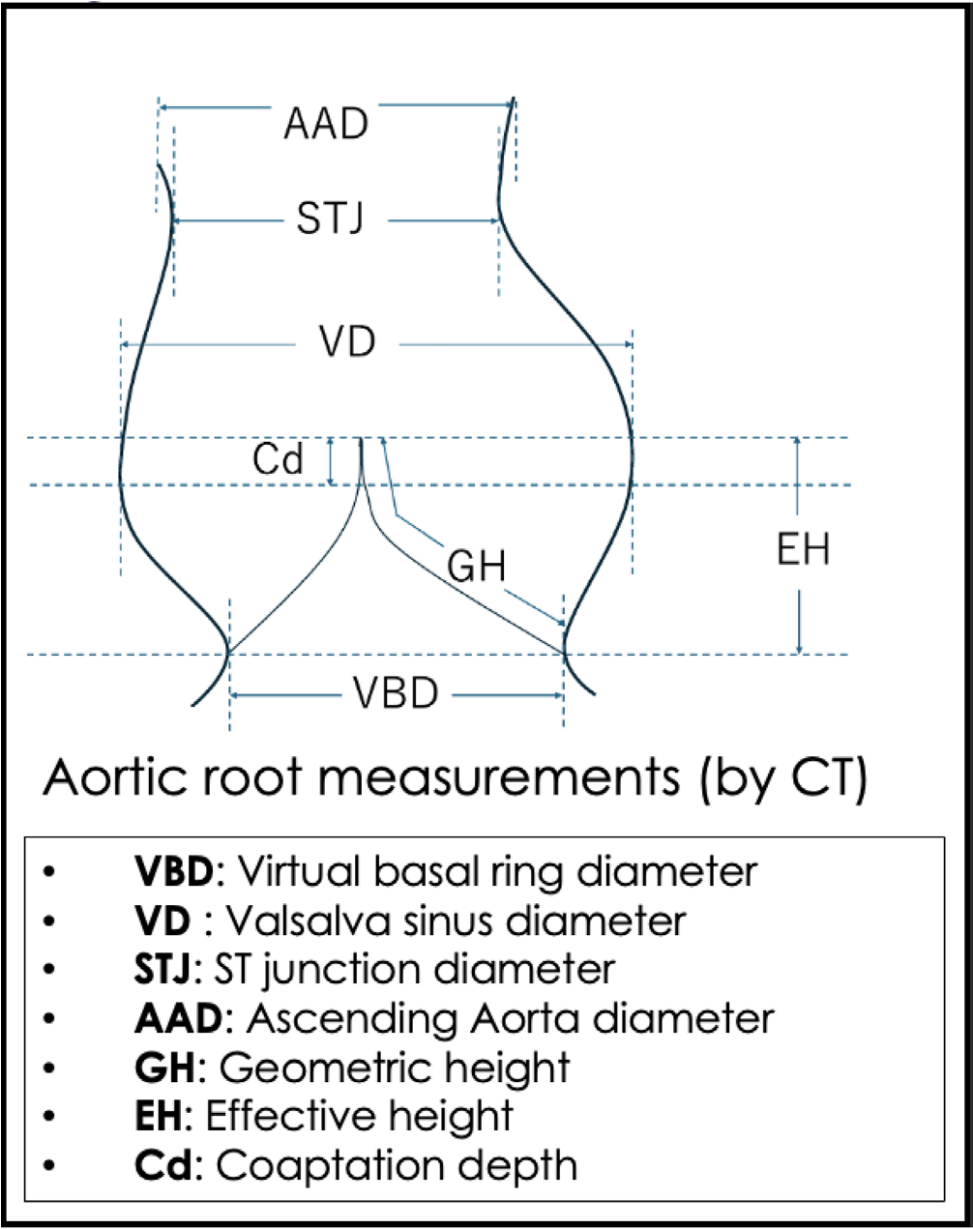
Aortic root morphology and definition of terms. [Color figure can be viewed at wileyonlinelibrary.com]

As shown in the Figure S1, 15 patients who underwent preoperative electrocardiography‐synchronized contrast‐enhanced cardiac computed tomography (CT) were included in the cohort. The patients were divided into two groups: those who developed significant AR during the follow‐up period after LVAD implantation (Group I, *n* = 10) and those whose AR was less than trivial (Group N, *n* = 5). Significant AR was defined as mild or greater AR. The morphology of the aortic root and valve (Figure 1) was measured using contrast‐enhanced cardiac CT, and avoidance rates of mild or greater AR were calculated. All data were retrospectively obtained from electronic medical records. The mean follow‐up period was 1208 ± 618 days after LVAD implantation for all patients.

### 2.1 Echocardiographic Evaluation and Measurements of Aortic Root and Valve Morphology

**FIGURE 2:**
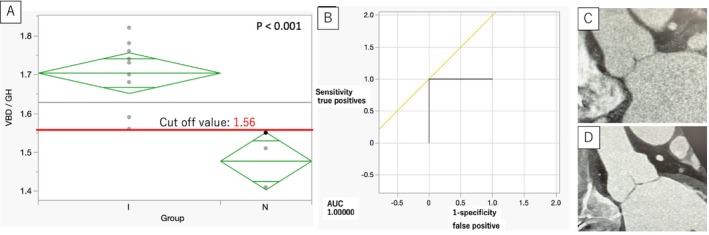
(A) One‐way ANOVA and t‐test for VBD/GH with the two groups and cutoff values shown using a box plot diagram, with diamonds indicating 95% confidence intervals. (B) ROC curve and the typical aortic root morphologies of (C) Group I and (D) Group N. [Color figure can be viewed at wileyonlinelibrary.com]

Comprehensive TTE was performed according to the American Society of Echocardiography [4]. The morphology of the aortic root and valve (Figure 2) was measured using a contrast‐enhanced cardiac CT scan, as previously described by Izawa et al. [5].

### 2.2 Statistical Analysis

All data analyses were performed using the JMP software (version 17.0; SAS Institute Inc., Cary, NC, USA). Data are expressed as mean ± standard deviation or median and range for continuous variables, as well as numerical values (percentages) for categorical variables. Continuous variables were compared using the unpaired *t*‐test.

The same analysis was performed using covariates considered to be associated with the development of de novo AI as represented in Table 2. The avoidance rate of AI was analyzed using the Kaplan–Meier method.

## 3 Results

### 3.1 Baseline Characteristics

We included 15 patients with a mean age of 52 years at the time of LVAD implantation. Among these, 73% were male, and 87% had nonischemic etiology. Cardiac functions and comorbidities before LVAD implantation were comparable between Group I and Group N (Table 1).

**TABLE 1:** Patient characteristics and preoperative data.

Preoperative	All (*n* = 15)	Group I (*n* = 10)	Group N (*n* = 5)	*p*
Patient characteristic				
Age	51.8 ± 13.3	51.9 ± 4.4	51.6 ± 6.2	0,97
Sex	11 (73.3%)	8 (80%)	3 (60%)	0,56
BSA (m^2^)	1.65 ± 0.19	1.64 ± 0.06	1.66 ± 0.09	0,86
Nonischemic cardiomyopathy	13 (86.7%)	8 (80%)	5 (100%)	0,52
Previous cardiovascular surgery	7 (46.7%)	5 (50%)	2 (40%)	1
Hypertension	2 (13.3%)	1 (10%)	1 (20%)	1
Dyslipidemia	3 (20%)	1 (10%)	2 (40%)	0,24
Diabates mellites	3 (20%)	2 (20%)	1 (20%)	1
COPD	0 (0%)	0 (0%)	0 (0%)	ー
Peripheral vascular disease	1 (6.7%)	1 (10%)	0 (0%)	1
CVA	1 (6.7%)	1 (10%)	0 (0%)	1
Hemoglobin (g/dl)	13.3 ± 2.27	12.8 ± 0.70	14.4 ± 0.99	0,21
White blood cell (/dl)	6203 ± 1800	6769 ± 524	5072 ± 742	0,084
LDH	211.6 ± 57.2	222.5 ± 18.0	189.8 ± 25.5	0,31
AST	24.3 ± 6.85	22.4 ± 2.0	28.2 ± 2.9	0,13
ALT	25.5 ± 14.7	22.6 ± 4.6	31.2 ± 6.5	0,3
T‐Bil	0.77 ± 0.32	0.88 ± 0.09	0.56 ± 0.13	0,069
Albumin	3.93 ± 0.41	3.78 ± 0.12	4.22 ± 0.16	0,046
Creatinine	1.01 ± 0.24	1.06 ± 0.074	0.898 ± 0.106	0,23
BNP	473.2 ± 332.7	485.0 ± 109.0	449.8 ± 154.2	0,86
TTE				
LVDd (mm)	67.5 ± 9.05	68.6 ± 2.93	65.4 ± 4.14	0,5388
LVDs (mm)	62.1 ± 9.40	63.4 ± 3.02	59.6 ± 4.27	0,4808
LVEF (%)	20.5 ± 7.88	20.1 ± 2.58	21.4 ± 3.65	0,7756
Trivial AI	6 (40%)	5 (50%)	1 (20%)	0,5804
MR	2.47 ± 1.41	2.70 ± 0.45	2.00 ± 0.63	0,3835
TR	2.00 ± 0.85	2.20 ± 0.26	1.60 ± 0.37	0,2059
INTERMACS level	2.53 ± 0.74	2.50 ± 0.24	2.60 ± 0.34	0,8162
RHC				
Heart rate	76.7 ± 13.1	78.6 ± 4.21	72.8 ± 5.95	0,4402
CVP (mmHg)	7.2 ± 4.72	7.40 ± 1.55	6.80 ± 2.19	0,8263
mPAP (mmHg)	27.5 ± 9.11	29.7 ± 2.79	23.0 ± 3.95	0,1894
PAWP (mmHg)	18.9 ± 8.35	20.8 ± 2.58	15.0 ± 3.65	0,2164
CI by Fick (L/min/㎡)	1.94 ± 0.76	1.91 ± 0.25	2.01 ± 0.35	0,819
CI by Thermo (L/min/㎡)	2.18 ± 0.66	2.27 ± 0.21	2.03 ± 0.30	0,5261
PVR (dyn・sec・cm‐5)	226.3 ± 113.8	226.4 ± 37.3	226.3 ± 52.8	0,9992
Preoperative therapy				
IMPELLA (pVAD)	0	0	0	ー
IABP	3 (20%)	1 (10%)	2 (40%)	0,2418
V‐A ECMO	0	0	0	ー
Ventilator	1 (6.7%)	0 (0%)	1 (20%)	0,3333
Catecholamines	13 (86.7%)	8 (80%)	5 (100%)	0,5238
HD	0	0	0	ー

Abbreviations: AI, Aortic insufficiency; ALT, Alanine aminotransferase; AST, Aspartate aminotransferase; BNP, Brain natriuretic peptide. BSA, Body surface area; CI, Cardiac index; COPD, Chronic obstructive pulmonary disease; CVA, Cerebrovascular accident; CVP, Central venous pressure; HD, Hemodialysis; IABP, Intra‐aortic balloon pumping; INTERMACS, Interagency registry for mechanically assisted circulatory support; LDH, Lactate dehydrogenase; LVDd, Left ventricular end‐diastolic diameter; LVDs, Left ventricular end‐systolic diameter; LVEF, Left ventricular ejection fraction; mPAP, Mean pulmonary artery pressure; MR, Mitral regurgitation; PAWP, Pulmonary artery wedge pressure; pVAD, Percutaneous ventricular assist device; PVR, Pulmonary vascular resistance; RHC, Right heart catheterization; T‐Bil, Total bilirubin; TR, Tricuspid regurgitation; TTE, Transthoracic echocardiography; V‐A ECMO, Venoarterial extracorporeal membrane oxygenation.

### 3.2 Preoperative Aortic Root and Valve Geometry

To define anatomical predictors of de novo AR, aortic valve and aortic root morphology were assessed (Table 2) using preoperative cardiac CT following previous literature [5]. The geometric height (GH) is measured for all left, right, and noncoronary cusps, respectively, and their average is calculated. The virtual basal ring diameter (VBD) measures the diameter at the level of the most left ventricular side of the cusps at end diastole. The VBD/GH in Group I (1.70 ± 0.024) was significantly greater than that in Group N (1.48 ± 0.034) (*p* = 0.0001) (Figure 2A). All other measurements were not significantly different between the two groups. The ROC curve was drawn for VBD/GH with a cutoff value of 1.56 to predict mild or greater de novo AR, with a sensitivity of 1.0000 and a specificity of 1.0000 (Figure 2B).

**TABLE 2:** Measurements of the aortic root and valve.

Preoperative aortic root and valve	All (*n* = 15)	Group I (*n* = 10)	Group N (*n* = 5)	*p*
Virtual basal ring diameter (mm)	25.5 ± 3.08	25.7 ± 1.00	25.1 ± 1.42	0,7577
Virtual basal ring diameter/BSA (mm/m^2^)	15.5 ± 2.01	15.8 ± 0.65	15.1 ± 0.92	0,5456
Valsalva diameter (mm)	31.4 ± 4.84	30.8 ± 1.56	32.7 ± 2.20	0,4833
Valsalva diameter/BSA (mm/m^2^)	19.2 ± 2.70	18.9 ± 0.86	20.0 ± 1.22	0,4662
ST junction diameter (mm)	26.1 ± 4.03	25.9 ± 1.32	26.3 ± 1.86	0,8605
ST junction diameter/BSA (mm/m^2^)	16.0 ± 2.24	15.9 ± 0.73	16.1 ± 1.03	0,829
Ascending aorta diameter (mm)	26.8 ± 4.74	26.3 ± 1.53	27.7 ± 2.17	0,6008
Ascending aorta diameter/BSA (mm/m^2^)	16.4 ± 2.45	16.1 ± 0.79	17.0 ± 1.12	0,5186
Geometric height (N) (mm)	16.5 ± 1.88	16.1 ± 0.59	17.1 ± 0.84	0,3608
Geometric height (L) (mm)	15.1 ± 2.29	14.3 ± 0.63	16.8 ± 0.89	0,0391
Geometric height (R) (mm)	15.0 ± 2.78	14.3 ± 0.84	16.4 ± 1.20	0,1767
Geometric height average (mm)	15.5 ± 1.92	14.9 ± 0.55	16.8 ± 0.79	0,0797
Geometric height average/BSA (mm/m^2^)	9.50 ± 1.07	9.15 ± 0.30	10.2 ± 0.43	0,0619
Effective height (N) (mm)	9.2 ± 1.37	9.2 ± 0.45	9.2 ± 0.63	1
Effective height (L) (mm)	8.5 ± 1.24	8.3 ± 0.77	8.9 ± 0.56	0,4378
Effective height (R) (mm)	9.3 ± 1.70	9.0 ± 0.53	10.0 ± 075	0,2915
Effective height average (mm)	9.0 ± 1.27	8.8 ± 0.41	9.3 ± 0.58	0,4783
Effective height average/BSA (mm/m^2^)	5.5 ± 0.57	5.4 ± 0.18	5.7 ± 0.25	0,328
Coaptation depth (N) (mm)	3.6 ± 0.65	3.5 ± 0.21	3.6 ± 0.30	0,7717
Coaptation depth (L) (mm)	3.4 ± 0.52	3.3 ± 0.16	3.6 ± 0.23	0,2944
Coaptation depth (R) (mm)	3.6 ± 0.65	3.6 ± 0.21	3.5 ± 0.30	0,7703
Minimum coaptation depth average (mm)	3.1 ± 0.51	3.1 ± 0.16	3.2 ± 0.23	0,6878
Maximum coaptation depth average (mm)	3.9 ± 0.45	3.9 ± 0.15	4.0 ± 0.21	0,91
Virtual basal ring/Geometric height	1.63 ± 0.13	1.70 ± 0.024	1.48 ± 0.034	0,0001

Abbreviations: BSA, body surface area; L, left coronary cusp; N, no coronary cusp; R, right coronary cusp; T, sinotubular junction.

### 3.3 Surgical Data and Initial Clinical Outcomes

No significant differences were found between the two groups for all items (Table 3).

**TABLE 3:** Operative data, early outcomes, and latest echocardiography.

	All (*n* = 15)	Group I (*n* = 10)	Group N (*n* = 5)	*p*
Operative data				
Operation time (min)	300.5 ± 74.9	321.6 ± 22.4	258.2 ± 31.6	0,1258
CPB time (min)	135.3 ± 44.4	148.2 ± 13.2	109 ± 18.7	0,1149
Cross clamp time	ー	ー	ー	ー
HeartMate 3	11 (73.3%)	6 (60%)	5 (100%)	0,2308
HeartMate 2	1 (6.7%)	1 (10%)	0 (0%)	1
HeartWare	2 (13.3%)	2 (20%)	0 (0%)	0,5238
Jarvik 2000	1 (6.67%)	1 (10%)	0 (0%)	1
Mitral procedure	0 (0%)	0 (0%)	0 (0%)	ー
Tricuspid procedure	2 (13.3%)	1 (10%)	1 (20%)	1
Early outcomes				
RVAD	2 (13.3%)	1 (10%)	1 (20%)	1
ECMO	1 (6.7%)	0 (0%)	1 (20%)	0,3333
Reexploration for bleeding	2 (13.3%)	2 (20%)	0 (0%)	0,5238
In hospital death	0 (0%)	0 (0%)	0 (0%)	ー
Latest TTE				
MR	1.47 ± 1.36	1.40 ± 0.44	1.60 ± 0.63	0,7988
TR	1.73 ± 0.70	2.00 ± 0.19	1.20 ± 0.27	0,0319
AR	1.73 ± 0.88	2.10 ± 0.23	1.00 ± 0.33	0,0163
Opening aortic valve	5 (33.3%)	2 (20%)	3 (60%)	0,2507

Abbreviations: AR, aortic regurgitation; CPB, cardiopulmonary bypass; ECMO, extracorporeal membrane oxygenation; MR, mitral regurgitation; RVAD, right ventricular‐assisted device; TR, tricuspid regurgitation; TTE, transthoracic echocardiography.

### 3.4 Postoperative Echocardiographic Data

According to the latest echocardiographic findings, there were no significant differences between Groups I and N in the degree of mitral regurgitation (Group I: 1.40 ± 0.44, Group N: 1.60 ± 0.63, *p* = 0.7988) and tricuspid regurgitation (Group I: 2.00 ± 0.19, Group N: 1.20 ± 0.27, *p* = 0.0319).

During the follow‐up period after LVAD implantation, an aortic valve was never opened in eight (80%) patients in Group I and two (40%) patients in Group N (*p* = 0.2507).

### 3.5 De Novo AR Avoidance Rate

**FIGURE 3:**
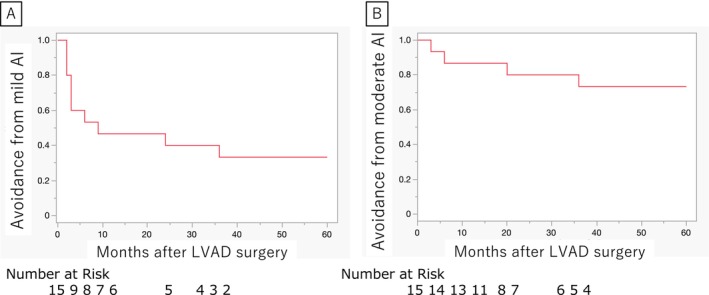
(A) Kaplan–Meier analysis showed avoidance rates for mild or greater aortic regurgitation (AR) of 50%, 42%, 40%, and 37% at 6, 12, 24, and 36 months, respectively, after left ventricular assist device (LVAD) implantation. (B) Kaplan–Meier analysis showed that the avoidance rates for moderate or greater AR were 80% and 73% at 24 and 36 months, respectively. [Color figure can be viewed at wileyonlinelibrary.com]

The long‐term de novo AR avoidance rates of the 15 patients were analyzed (Figure 3). The avoidance rates for mild or greater AR were 50%, 42%, 40%, and 37% at 6, 12, 24, and 36 months, respectively. The avoidance rates for moderate or severe AR were 80% and 73% at 24 and 36 months, respectively.

## 4 Discussion

Changes in aortic hemodynamics after LVAD implantation, older age, no aortic valve opening, smaller Valsalva sinus size, as well as lower left ventricular and aortic volumes have been reported as independent predictors of developing AR after LVAD implantation [6, 7, 8, 9, 10]. However, the relationship between the detailed morphology of the aortic root and de novo AR after LVAD implantation has scarcely been described. In the present study, a large VBD/GH was significantly associated with the occurrence of de novo AR, and although further studies on a larger number of cases are needed to confirm the present findings, the cutoff value of VBD/GH of 1.56 may be one indicator of whether or not to perform surgical intervention on the aortic valve when performing LVAD implantation surgery. Several studies have suggested that dilatation of the proximal ascending aorta [3] or a combination of the ST junction and proximal ascending aortic dilatation may be associated with the development of significant AR [11, 12]. Nishida et al. reported that dilatation of the proximal ascending aorta was a risk factor for de novo AR [3]. In their study, patients with de novo AR were significantly older than those without, and multivariable analysis failed to show an association between age and the development of de novo AR, although age‐related changes in aortic morphology can be taken into account. In the present study, there was no significant difference in age between the AR and non‐AR groups, which allowed us to compare aortic morphology without age‐related considerations. Prior literature has also shown that Cd and EH are the key factors for preventing AR; shorter Cd or shorter EH has been associated with the development of AR [11, 12, 13, 14]. The VBD/GH ratio, representing the length of aortic valve leaflet relative to aortic annulus, potentially relates to coaptation depth after increased downward pressure with continuous‐flow LVAD. Although further studies are needed, the ratio of VBD/GH can be a valuable parameter to predict de novo AR, simultaneously considering the anatomy of both the aortic root and valve leaflet.

### 4.1 Limitations

There are some limitations that should be addressed in this study. First, this was a single‐center, retrospective study. Selection bias and confounding factors cannot be completely excluded. Second, the sample size of this cohort study was small.

## 5 Conclusions

In conclusion, a greater aortic VBD/GH before LVAD surgery was associated with the occurrence of significant de novo AR after LVAD implantation.

## Author Contributions

Takashi Murakami, Yusuke Misumi, and Daisuke Yoshioka contributed to the study's conception and design. Takuji Kawamura contributed to the critical revision of the article. Ai Kawamura, Shin Yajima, Shunsuke Saito, and Takashi Yamauchi contributed to data acquisition. Shigeru Miyagawa contributed to the approval of the article. Takashi Murakami drafted, and all authors revised the manuscript.

## Conflicts of Interest

The authors declare no conflicts of interest.

## Supporting information


**Figure S1.** Consecutive patients (*n* = 87) who underwent LVAD implantation at our institution between 2018 and 2023 were reviewed. Twenty‐three patients who underwent concurrent aortic valve surgery were excluded. Among the remaining 64 patients, 55 had no or trivial AI on preoperative transthoracic echocardiography (TTE). Of these, 15 patients who underwent preoperative electrocardiography‐synchronized contrast‐enhanced cardiac computed tomography (CT) were included in the cohort (HeartMate 3: *n* = 11, HeartMate II: *n* = 2, and HeartWare: *n* = 2). The patients were divided into two groups: those who developed significant AR during the follow‐up period after LVAD implantation (Group I, *n* = 10) and those whose AR was less than trivial (Group N, *n* = 5). Significant AR was defined as mild or greater AR.
