# Supplementary material for: Association of Aortic Root and Valve Morphology With De Novo Aortic Valve Regurgitation After Implantation of Left Ventricular Assist Device
Source: Artif Organs. 2025 Mar 12;49(8):1339–45. doi: 10.1111/aor.14987 (PMC12269341; doi:10.1111/aor.14987)
Supplement: Supplementary file 1 — Figure S1. Consecutive patients (n = 87) who underwent LVAD implantation at our institution between 2018 and 2023 were reviewed. Twenty‐three patients who underwent concurrent aortic valve surgery were excluded. Among the remaining 64 patients, 55 had no or trivial AI on preoperative transthoracic echocardiography (TTE). Of these, 15 patients who underwent preoperative electrocardiography‐synchronized contrast‐enhanced cardiac computed tomography (CT) were included in the cohort (HeartMate 3: n = 11, HeartMate II: n = 2, and HeartWare: n = 2). The patients were divided into two groups: those who developed significant AR during the follow‐up period after LVAD implantation (Group I, n = 10) and those whose AR was less than trivial (Group N, n = 5). Significant AR was defined as mild or greater AR. [file AOR-49-1339-s001.pdf]

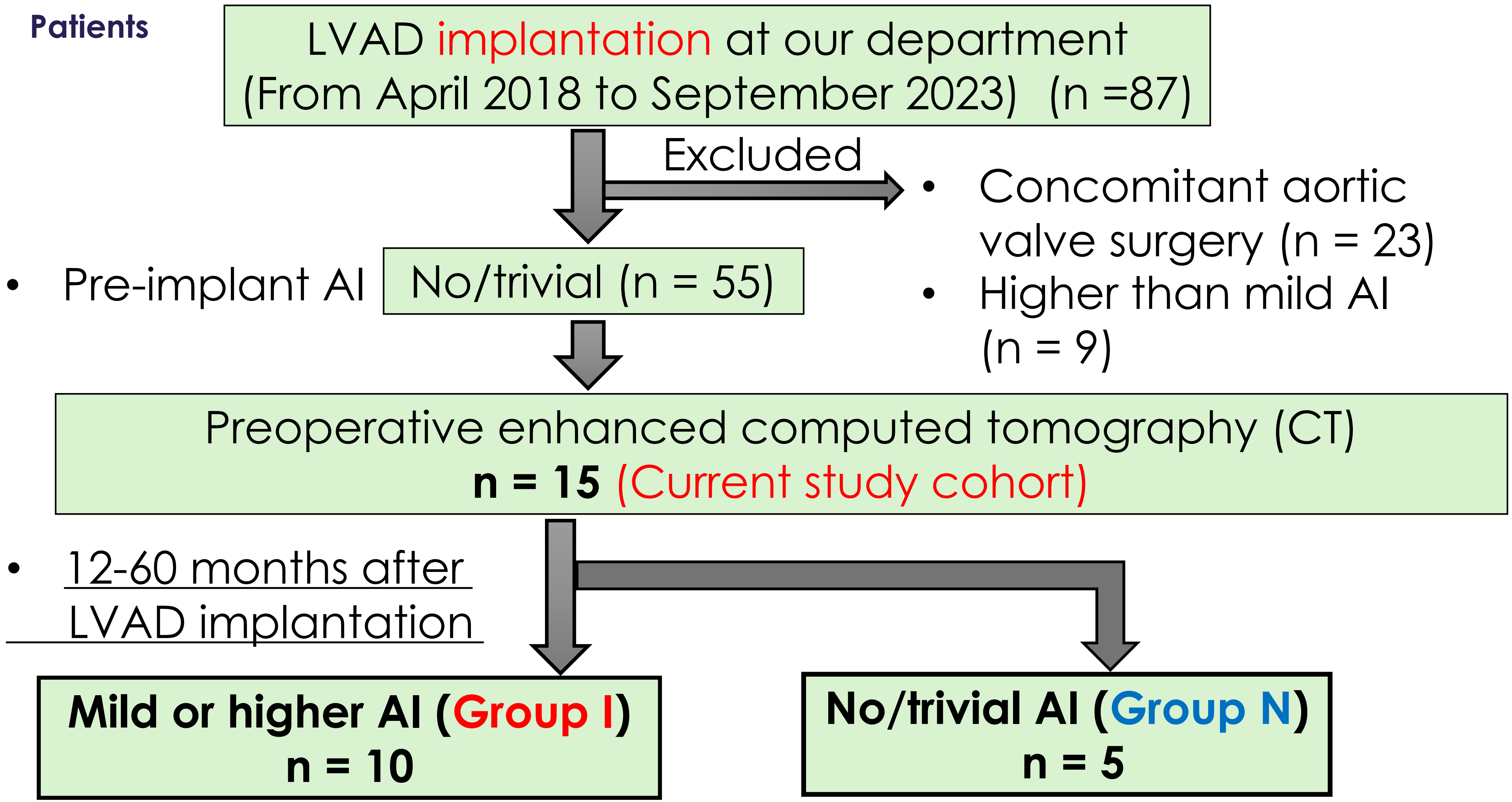
Patients
LVAD implantation at our department
(From April 2018 to September 2023) (n =87)
Excluded
• Concomitant aortic valve surgery (n = 23)
• Higher than mild AI (n = 9)
• Pre-implant AI
No/trivial (n = 55)
Preoperative enhanced computed tomography (CT)
n = 15 (Current study cohort)
• 12-60 months after LVAD implantation
Mild or higher AI (Group I)
n = 10
No/trivial AI (Group N)
n = 5
